# Comprehensive overview of the global pharmacogenetic and functional variability in *NUDT15*

**DOI:** 10.1186/s40246-026-00959-2

**Published:** 2026-04-04

**Authors:** Yitian Zhou, Lukas M. Lauschke, Volker M. Lauschke

**Affiliations:** 1 Department of Physiology and Pharmacology and Center for Molecular Medicine, Karolinska Institutet and University Hospital, Stockholm, Sweden; 2 Dr. Margarete Fischer-Bosch Institute of Clinical Pharmacology, Stuttgart, Germany; 3 University of Tübingen, Tübingen, Germany; 4 Department of Pharmacy, The Second Xiangya Hospital, Central South University, Changsha, China

**Keywords:** Allele frequency, Thiopurine, Population pharmacogenomics, Metabolizer phenotype, Precision medicine

## Abstract

Thiopurine medications are widely used for the treatment of acute lymphoblastic leukemia and inflammatory bowel disease. However, thiopurine-induced hemotoxicity occurs in up to 30% of patients, which causes substantial morbidity and limits therapeutic success. These adverse effects are associated with genetic variation in *TPMT* and *NUDT15*, which encode key enzymes involved in thiopurine metabolism. While the global distribution of *TPMT* variability has been extensively studied, less is known about the ethnogeographic variability of *NUDT15,* and no meta-analysis of available population frequencies has been reported. In this study, we aggregated genetic data from 150 studies and repositories comprising genotypes from a total of 1,401,592 individuals across 55 countries. We further synthesize the available clinical evidence for functional effects of NUDT15*5 and NUDT15*6, which are currently considered as variants with indeterminate functional effects. This synthesis indicates that both alleles significantly increase thiopurine adverse event risk. We aggregate the genetic information to spectra of functional variability to generate the first global maps of NUDT15 metabolizer phenotypes. We observe a graded distribution of NUDT15 deficiency, peaking in East Asia (46% of all individuals exhibit reduced NUDT15 activity) and declining across Central Asia (13.6%), Middle Eastern populations (3.8%) and Europe (2.1%). NUDT15 deficiency is furthermore prevalent on the Western coast of South and Central America but absent in Africa. Overall, our results provide a valuable resource for population-specific *NUDT15* allele and metabolizer phenotype distributions, which can guide thiopurine risk assessment on a global scale.

**Supplementary Information:**

The online version contains supplementary material available at 10.1186/s40246-026-00959-2.

## Introduction

Thiopurine medications, including 6-mercaptopurine, 6-thiogranine and azathioprine, are commonly prescribed for the treatment of acute lymphoblastic leukemia (ALL) and inflammatory bowel disease (IBD). Thiopurines are pro-drugs that are metabolically activated by thiopurine S-methyltransferase (TPMT) into 6-thioguanine nucleotides (6-TGNs). Further metabolism of 6-TGNs generates 6-thioguanosine triphosphate (6-TGTP) and 6-thiodeoxyguanosine triphosphate (6-TdGTP), which can be incorporated into DNA or RNA, thereby inducing cell death. This activation process is counterbalanced by nudix hydrolase 15 (NUDT15), which limits thiopurine toxicity by hydrolyzing the triphosphate thereby regenerating the monophosphatic forms [20]. Importantly, dysregulation of this metabolic equilibrium can result in excessive accumulation of active cytotoxic metabolites, which can manifest as dose-limiting adverse effects, primarily manifesting as myelotoxicity and hepatotoxicity, in 10–30% of thiopurine-treated patients [5, 12].

Genetic variability has been recognized as an important determinant of thiopurine toxicity. In particular, there are multiple variations in the *TPMT* gene, particularly the TPMT*2, *3A and *3C alleles that cause a significant reduction in TPMT activity, thereby shunting thiopurine metabolism toward cytotoxic metabolites [33]. However, *TPMT* variation alone accounts for only approximately 25% of myelosuppression cases [8]. Especially in Asian populations, *TPMT* variants are less common and thus cannot explain the prevalence of thiopurine-induced leukopenia, which is even higher than in European populations [3, 7, 34]. Subsequent genome-wide association studies (GWAS) identified a variant in *NUDT15* p.Arg139Cys (rs116855232), that was significantly associated with thiopurine-induced leukopenia [31, 32]. Critically, this variant abolishes enzyme function by destabilizing the catalytic site of the NUDT15 enzyme, resulting in elevated levels of cytotoxic thiopurine triphosphate metabolites [16]. Further genetic analyses identified four coding variants in *NUDT15*, which individually defined the star alleles NUDT15*3 (p.Arg139Cys), *4 (p.Arg139His), *5 (p.Val18Ile) and *6 (p.Val18_Val19insGlyVal). These variants reduced NUDT15 activity by 74–100% of wild-type levels and were significantly associated with thiopurine intolerance across cohorts from Guatemala, Singapore and Japan [19].

Given the clinical significance of functional NUDT15 variability for thiopurine treatment outcomes, understanding the global distribution of key *NUDT15* alleles is critical for population-stratified genomic medicine. However, current reports are either limited to one or few individual populations or evaluate *NUDT15* variant frequencies in highly aggregated superpopulations [20, 23]. Here, we thus conducted a comprehensive synthesis of functionally important *NUDT15* alleles encompassing a total of 1,401,592 individuals across 55 countries and translate the resulting frequencies into country-specific metabolizer phenotype distributions. These results provide the first comprehensive high-resolution landscape of pharmacogenetic variability in *NUDT15*, which constitutes a relevant resource to inform genetically guided thiopurine therapy on a global scale.

## Results

### Geographic distribution of functionally important *NUDT15* alleles

**Fig. 1:**
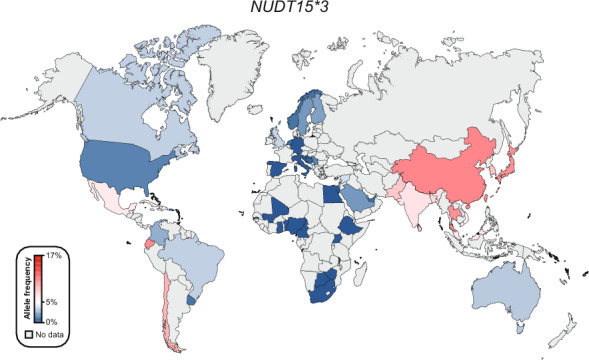
Global distribution of NUDT15*3 allele. Frequencies of 55 countries were color-coded with the highest frequency in red and the lowest frequency in blue. Countries with no frequency information available are colored grey

We conducted our analysis on seven *NUDT15* star alleles (*3-*9) included in the Clinical Pharmacogenetics Implementation Consortium (CPIC) guideline [23]. NUDT15*2 as an allele is obsolete since it was recently consolidated with NUDT15*3 and redesignated as *3.002. Frequencies of NUDT15*3 are most commonly investigated with information being available for 55 countries (Table 1, Fig. 1; Supplementary Table 1). The highest frequencies for NUDT15*3 were observed in East Asian populations, ranging from 6.8% and 7.6% in Malaysia and Vietnam to 17% in Singapore. In Central and South Asian populations, the frequencies are slightly lower, pivoting around 7–8%. In contrast, NUDT15*3 is less common in Middle Eastern, where minor allele frequencies (MAFs) range between 0% and 0.3% in Egypt and Jordania to 3.6% and 3.8% in Lebanon and Syria, respectively. In Europe, NUDT15*3 is overall rare with the highest frequencies being surprisingly reported for Finland (MAF = 2.1%) and Sweden (MAF = 2%). These results indicate a graded distribution of NUDT15*3 from East Asia (5.6–17%) over Central Asia (6.8–8.3%) to the Middle East (0.3–3.8%) and Europe (0–2.1%). Interesting, we observed substantial variability in the frequency of NUDT15*3 across American populations. The allele is relatively abundant along the west coast of South America (Peru MAF = 11.8%; Chile MAF = 9.5% and Mexico MAF = 6.5%). NUDT15*3 is not detected in any of the 13 African countries for which frequency information was available.

**Table 1:** Country-specific frequencies of major *NUDT15* alleles

Country	N (individuals)	*3 (in %)	*5 (in %)	*6 (in %)	*9 (in %)
Europe					
Croatia	522	0.4	0	0	0
England	201,113	0.4	0.01	0.3	0.2
Finland	32,004	2.1	0	1.3	0.08
Germany	689	0	–	0	–
Italy	566	0.5	–	–	1.2
Netherlands	988	0.7	–	–	–
Norway	2,855	0.9	0	0	0.3
Scotland	91	0	–	–	–
Serbia	113	1.3	0	0	0
Slovenia	146	0.5	–	–	–
Spain	107	0	–	–	–
Sweden	102	2	–	–	–
Sub-Saharan Africa					
Benin	50	0	–	–	–
Botswana	47	0	–	–	–
Burkina Faso	33	0	–	–	–
Cameroon	26	0	–	–	–
Ethiopia	142	0	–	0	–
Gambia	113	0	–	–	–
Ghana	26	0	–	–	–
Kenya	99	0	–	–	–
Nigeria	260	0	–	–	–
Sierra Leone	85	0	–	–	–
South Africa	157	0	–	–	–
Uganda	1,989	0	0	0	0
Zambia	41	0	–	–	–
North Africa and Middle East					
Egypt	150	0	–	–	–
Jordan	161	0.3	0	0	–
Lebanon	273	0.4	–	–	–
Qatar	6,045	2.1	–	–	–
Saudi Arabia	11,889	1.8	0.1	–	–
Syria	92	3.8	–	–	–
United Arab Emirates	100	0.5	–	–	–
Central and South Asia					
Bangladesh	270	7.2	–	–	–
India	4,341	6.9	–	–	–
Pakistan	96	8.3	–	–	–
Sri Lanka	670	8.3	–	–	–
East Asia					
China	702,171	11.5	1.3	4.1	1.1
Japan	8,828	13.6	1.2	0.4	0.06
Malaysia	140	6.8	0.7	–	–
Singapore	329	17	1	4	–
South Korea	2,792	11	1.1	0.8	–
Thailand	353	10.5	1.2	4.1	–
Vietnam	269	7.6	–	3.2	–
North America					
Canada	1,083	3.3	–	–	–
U.S.	415,729	1.3	0.03	1.04	0.1
Central and South America					
Barbados	96	0	–	–	–
Brazil	544	3.5	–	–	–
Chile	324	9.5	–	–	–
Colombia	162	2.5	–	–	–
Guatemala	144	2	–	–	–
Mexico	1,270	6.5	–	–	–
Peru	85	11.8	–	–	–
Puerto Rico	104	0.5	–	–	–
Uruguay	115	3	–	1.3	–
Oceania					
Australia	603	3.1	–	–	–

In addition to NUDT15*3, NUDT15*5 and NUDT15*6 were also found to be common in East Asian populations, with frequencies ranging from 0.7 to 4.1%. MAFs of NUDT15*6 were 3–4% across East Asia with the exception of the Japanese and South Korean population where the allele was rare (0.4–0.8%). Due to the lack of reported frequency data for Central Asian populations, it remains how these alleles are distributed outside of Southeast Asia. In the Americas, NUDT15*6 is observed primarily in Uruguay (MAF = 1.3%) and the USA (MAF = 1%). Among the remaining alleles, only NUDT15*9 has been reported at frequencies above 1% in Chinese (MAF = 1.1%) and Italian (MAF = 1.2%) populations, whereas NUDT15*4 and NUDT15*7 are rare worldwide (Table 1; Supplementary Table 1).

### Evaluation of the functional impact of NUDT15*5 and NUDT15*6

**Fig. 2:**
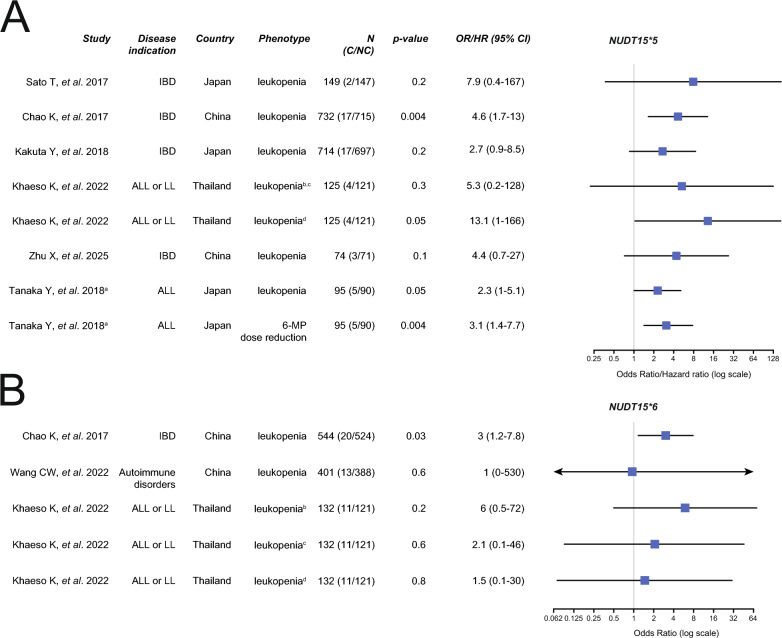
Clinical impact of NUDT15*5 and *6 alleles on thiopurine treatment response. Forest plots display quantitative effect estimates from clinical studies for NUDT15*5 (**A**) and *6 (**B**) alleles. ^a^Hazard ratio was used for quantitative effect measurement; ^b^Grade 4 neutropenia at the first 2 months of the maintenance phase; ^c^Grade 4 neutropenia at the first 4 months of the maintenance phase; ^d^Grade 4 neutropenia at the first 6 months of the maintenance phase

Based on the currently available guidelines, NUDT15*3 is well recognized as a loss-of-function allele, while the functional impacts of NUDT15*4, *5 and *6 are considered unclear [23]. In vitro, the intrinsic clearance (CL_int_) of NUDT15*3 is zero, while CL_int_ for NUDT15*4, *5 and *6 is 23.6%, 27.7% and 14.9% of wildtype (*1), respectively [19]. To evaluate the clinical impacts of these alleles, we conducted a comprehensive literature survey. The available clinical studies were highly heterogeneous and differed across disease indication, country and type of adverse event. Six studies were included to evaluate the clinical impact of NUDT15*5, comprising a total of 45 carriers and 1888 controls (Fig. 2A). Notably, we observed a clear trend towards increased risk and likelihood for dose reduction among NUDT15*5 carriers with individual odds ratios (OR) for allele carriers ranging from 2.7 to 13.1 and three studies individually reaching statistical significance (p < 0.05). For NUDT15*6, three studies were identified, including a total of 44 carriers and 1033 controls (Fig. 2B). Similarly to NUDT15*5, the available studies suggested an overall increased risk of thiopurine-induced leukopenia in NUDT15*6 carriers (OR up to 5.95), although only one study reaching statistical significance. NUDT15*4 was too rare to evaluate its functional status. Based on the available clinical evidence, we concluded both NUDT15*5 and *6 have clinically meaningful impacts on enzyme function and confer an increased risk of thiopurine-induced adverse reactions.

### Global distribution of *NUDT15* phenotypes

**Fig. 3:**
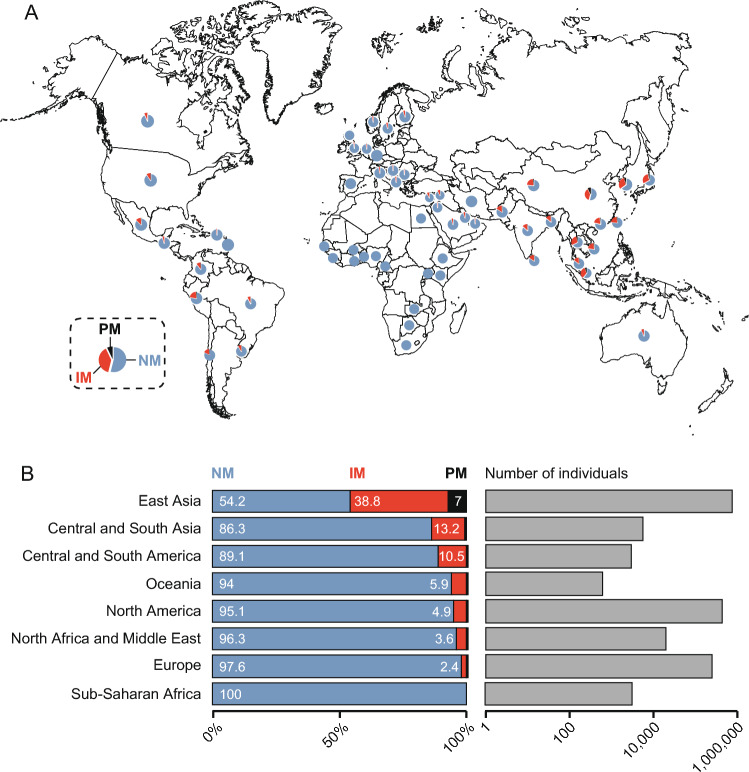
NUDT15 metabolizer phenotypes across different countries and ethnogeographic groups. **A** Pie charts illustrate the percentage of normal metabolizer (NM, in blue), intermediate metabolizer (IM, in orange) and poor metabolizer (NM, in black) for representative countries and regions. **B** Frequencies of NUDT15 NM, PM, and IM (in %) across different ethnogeographic groups (left), and the number of individuals analyzed for each group (right)

Next, we inferred the global spectrum of NUDT15 functionality. To this end, we aggregated country-specific frequencies of the loss-of-function allele NUDT15*3, with NUDT15*5 and *6 due to the aforementioned substantial evidence for an increase in leukopenia risk (Fig. 3; Table 2). East Asian populations harbored the highest fraction of NUDT15 intermediate metabolizers (IM; 38.8%) and are the only superpopulation in which a substantial number of poor metabolizers (PM) are observed (0.6–7.1%). Notably, nearly half of the population in the central and eastern regions of China carries at least one *NUDT15* risk allele. The prevalence of IM and PM decreases markedly toward Central and South Asia (< 16%) and becomes even lower in the Middle Eastern (< 8%) and European (< 7%) populations. The Americas represent another hotspot for reduced NUDT15 metabolism, with IM and PM prevalence reaching 22.1% in Peru and 18% in Chile. While *NUDT15* risk allele carriers are relatively common across the Americas, low frequencies of IM and PM are observed in the Caribbean territories, such as Puerto Rico (1%) and Barbados (0%), likely due to the high frequency of African origin. Interestingly, Australia exhibits a combined prevalence of 6% IM and PM, which is higher than that reported in European populations. No frequency information is available for other Oceanian populations.

**Table 2:** Frequencies of inferred NUDT15 metabolizer phenotype

Country	NM (in %)	IM (in %)	PM (in %)
Europe			
Croatia	99.2	0.8	< 0.01
England	98.3	1.7	< 0.01
Finland	93.2	6.7	0.1
Germany	100	0	0
Italy	97.6	2.4	< 0.1
Netherlands	98.7	1.3	< 0.01
Norway	97.6	2.4	< 0.1
Scotland	100	0	0
Serbia	97.4	2.6	< 0.1
Slovenia	99	1	< 0.01
Spain	100	0	0
Sweden	96.1	3.9	< 0.1
Sub-Saharan Africa			
Benin	100	0	0
Botswana	100	0	0
Burkina Faso	100	0	0
Cameroon	100	0	0
Ethiopia	100	0	0
Gambia	100	0	0
Ghana	100	0	0
Kenya	100	0	0
Nigeria	100	0	0
Sierra Leone	100	0	0
South Africa	100	0	0
Uganda	100	0	0
Zambia	100	0	0
North Africa and Middle East			
Egypt	100	0	0
Jordania	99.4	0.6	< 0.01
Lebanon	99.3	0.7	< 0.01
Qatar	95.8	4.1	0.1
Saudi Arabia	96.4	3.6	< 0.1
Syria	92.5	7.3	0.2
United Arab Emirates	99	1	< 0.01
Central and South Asia			
Bangladesh	86.1	13.4	0.5
India	86.6	12.9	0.5
Pakistan	84	15.3	0.7
Sri Lanka	84.1	15.2	0.7
East Asia			
China	53.9	39	7.1
Japan	71.8	25.9	2.3
South Korea	75.9	22.5	1.7
Malaysia	85.5	13.9	0.6
Singapore	60.9	34.3	4.8
Thailand	67.9	29	3.1
Vietnam	79.5	19.3	1.2
North America			
Canada	93.5	6.4	0.1
USA	95.1	4.9	< 0.1
Central and South America			
Barbados	100	0	0
Brazil	92.6	7.2	0.2
Chile	82	17.1	0.9
Colombia	89.5	10.2	0.3
Guatemala	95.9	4.1	< 0.1
Mexico	87.4	12.2	0.4
Peru	77.9	20.7	1.4
Puerto Rico	99	1	< 0.01
Uruguay	91.5	8.3	0.2
Oceania			
Australia	94	5.9	0.1

*NM* normal metabolizers, *IM* intermediate metabolizers, *PM* poor metabolizers

## Discussion

Genetic variation in *NUDT15* is recognized as a population-specific predictor of thiopurine-induced toxicity. Here, we comprehensively profiled the global distribution of functionally important NUDT15 alleles, covering all pharmacogenetic biogeographic groups [11]. Country-specific allele frequency profiling is particularly valuable because it reveals granular population differences that cannot be captured by analyses at the level of superpopulations. Furthermore, such frequencies provide pharmacogenomic evidence that can be directly leveraged to guide healthcare policy decisions, which are typically made at the national level. This approach may however encounter limitations when analyzing countries such as the U.S. or Brazil, which have a highly admixed population that cannot be easily categorized into well-defined ethnic groups [27]. The *NUDT15* variant frequencies in the U.S. analyzed in this study were from nationwide sequencing projects, such as the All of Us Research Program, the 1000 Genomes Project and the Genome Aggregation Database, as well as from clinical studies including patients of diverse ethnic backgrounds, including Hispanic, European, African, East Asian, and indigenous populations. While frequencies from these studies reflect the overall risk in the general U.S. population, they do not capture ancestry specificity of *NUDT15* alleles. Consequently, population-level estimates may underestimate the risk for the East Asian subpopulations while overestimating the risk for others. Similarly, *NUDT15* variant frequencies in the Brazilian population have been reported in multiple studies analyzing indigenous groups in the Brazilian Amazon [9, 22, 24]. The results shows that there was substantial variation in NUDT15*3 frequencies, which range from 0.9 to 7.2%. Therefore, higher resolution data that consciously includes also indigenous populations is of high importance for pharmacogenomic-guided precision medicine.

Based on country-specific frequencies of functional *NUDT15* variant alleles (*3, *5, *6, and *9), we inferred the distribution of NUDT15 metabolizer phenotypes (NM, IM and PM) across 55 countries. Although NUDT15*3 frequency data were available for all countries included in the analysis, data for *5 (12 countries), *6 (15 countries) and *9 (9 countries) was more limited. The absence of frequency data for certain alleles in key populations may considerably influence the accuracy of predicted metabolizer phenotype frequencies. For example, the substantially lower frequencies of IM and PM in Malaysia compared with other East Asian countries might be attributable to the absence of frequency data for NUDT15*6, an allele that occurs at approximately 3–4% in most East Asian populations. In addition, given the similarly high NUDT15*3 frequencies observed between Central/South Asian and East Asian populations, it is plausible that the currently missing Central and South Asian frequency data for NUDT15*6 are also comparable to those reported in East Asian populations. Likewise, although NUDT15*6 frequency data are lacking for most countries in the Americas, it can be hypothesized that they are similar to those reported in Uruguay and the USA, at approximately 1%. Therefore, it is important to note that the current metabolizer phenotype estimates provided here are conservative and the true prevalences may be higher if further alleles are included.

The occurrence of adverse events during thiopurine treatment is influenced by genetic variability in both *TPMT* and *NUDT15*. The global distribution of functionally important *TPMT* alleles, including TPMT*2, *3A and *3C, have been reported previously [10, 17]. TPMT*3A is most prevalent in American and European populations, with average frequencies above 3%, and the highest frequency observed in the Montubio ethnic group in Ecuador, reaching 16% [10, 29]. In contrast, TPMT*3C is generally rare in the Americas and Europe, but is relatively common in some Asian and African countries, such as India (MAF = 2.3%) [15], Thailand (MAF = 5%) [26], Mozambique (MAF = 3.8%) [21], Kenya (MAF = 5.4%) [18], and Ghana (MAF = 7.6%) [1]. TPMT*2 is observed only in certain Middle Eastern and Southern European populations, with frequencies around 1%, and is absent in other regions of the world [29]. This information, combined with the global distribution of functional *NUDT15* alleles reported in this study, suggests that thiopurine toxicity is predominantly determined by *TPMT* variability in Europe and Africa, whereas *NUDT15* is the key determinant in East Asian populations. In Central and South America, both *TPMT* and *NUDT15* risk alleles should be considered to estimate thiopurine risk.

## Conclusions

In summary, this study provides the first global frequency landscape for major *NUDT15* alleles. Aggregation of the genetic data into high-resolution maps of metabolizer phenotypes reveals a clear distribution pattern of NUDT15 deficiency, identifying Asian and American populations as distinct high-risk groups. Combined with functional genetic variation in *TPMT*, this resource offers important evidence to guide pharmacogenetic-informed optimization of thiopurine therapy, thereby supporting precision medicine and improving healthcare at a global scale.

## Methods

### Literature survey and data integration

We performed a comprehensive literature search to identify studies reporting frequencies of *NUDT15* star alleles. The databases PubMed and Google Scholar were searched up to November 2025 using combinations of the following terms: “NUDT15”, “NUDT15 polymorphism”, “NUDT15 variant”, “NUDT15 allele”, “NUDT15 star allele”, “NUDT15 frequency”, and “thiopurine”. Reference lists of relevant publications were also screened to identify additional studies. Only studies were included that (i) reported population-level frequencies of *NUDT15* variants or star alleles, (ii) provided sufficient information to derive allele frequencies, and (iii) were published in English. Studies focusing exclusively on functional characterization without population frequency data, case reports, reviews or duplicate datasets were excluded. When multiple publications reported data from the same cohort, only one instance was included. Two investigators independently screened titles and abstracts, followed by full-text review of potentially eligible articles. Data extracted included study population, country, sample size and reported *NUDT15* allele frequencies. In addition to published studies, we included population frequency data from the Genome Aggregation Database (gnomAD) [6], the Phase 3 1000 Genomes Project [2], the All of Us Research Program (2024) and the Norwegian Variant Frequency Database (https://variant.norgene.no/). Since gnomAD also includes data from the 1000 Genomes Project, we considered only those data that avoided overlap. As a result, we identified a total of 150 data sources reporting *NUDT15* allele frequencies from 1,401,592 individuals (Supplementary Table 2). Frequency data for countries and ethnogeographic groups were aggregated using a weighted average approach using the cohort sizes as weighting factors. Furthermore, seven clinical studies were identified [4, 13, 14, 25, 28, 30, 35] to evaluate the impact of NUDT15*5 and *6 on thiopurine-induced adverse reactions.

### Genotype to phenotype translations

NUDT15*3 was classified as risk allele based on the CPIC guideline [23] NUDT15*5 and *6 are considered risk alleles based on the results presented in this study. Frequencies of these alleles were aggregated and used to calculate phenotype distributions based on the Hardy–Weinberg equation.

## Supplementary Information

Supplementary material 1. Table 1. Aggregated country-specific *NUDT15* allele frequencies with 95% confidence intervals and 95% prediction intervals.

Supplementary material 2. Table 1. Frequencies of *NUDT15* alleles reported by different research studies and genomic databases.

## Data Availability

Frequency information from all considered studies is provided in the Supplement.
